# Learning Microvascular Anastomosis in Low Socioeconomic Vascular Models During Residency

**DOI:** 10.7759/cureus.1199

**Published:** 2017-04-27

**Authors:** Karuna Tamrakar

**Affiliations:** 1 Neurosurgery, College of Medical Sciences, Bharatpur, Nepal

**Keywords:** umbilical cord, chicken wings, neurosurgical training, microvascular anastomosis

## Abstract

Microvascular anastomosis procedure has become an essential practice for the management of most neurovascular diseases. Increasing use of neurosurgical techniques necessitates intensive laboratory training in microsurgery.

Umbilical artery is used for quantifiable representation to set up microvascular anastomosis model for the beginners. These arteries are found to be between 4 and 5 mm in diameter. Chicken wings are set up as second anastomosis model. Five to six centimeter long brachial artery extracted from a chicken is measured approximately 1-2 mm in diameter. These arteries are practiced for end-to-end, end-to-side, or side-to-side anastomosis under the microscope.

Umbilical cord and chicken wing model hold several advantages. These essentials are inexpensive, convenient to manage, and easy to obtain for educational purposes. They neither need detailed facilities for maintenance like in animal model nor any anesthetic prerequisite. Moreover, the diameter and structure of the material are identical to those of human cortical vessels.

Low-cost laboratory training during residency is more relevant in source restraint areas. It has several added benefits in refining the procedural dexterity on anastomosing smaller size vessel identical to a cortical vessel of middle cerebral artery and distal branches of the superficial temporal artery.

## Introduction

Laboratory practice has proven to be a necessary preparation along with patient care and surgical skills. However, it is not yet popular among the surgical residents in developing countries. Microsurgical procedure for bypass surgery is a challenging subspecialty of neurosurgery. It has been an essential procedure for the management of occlusive cerebrovascular diseases for revascularization and bypass surgery when collateral circulation is inadequate in substituting blood flow after permanent vessel occlusion. Yasargil, in 1967 [1], first put forth the hypothesis of cerebral revascularization. The concept and techniques for bypass surgeries have evolved since for reconstruction of both the anterior and posterior circulations. Various bypass techniques have been described [2]. In current practice, extracranial to intracranial bypass is performed for flow augmentation in ischemic condition. This requires good dexterity and technical skill. Modest microsurgical skill affects the learning curve of a vascular neurosurgeon and may eventually risk patients in terms of morbidity and mortality, which signifies necessity of proper microvascular training before handling patients in operation theater. Despite its demand in the current management of complex cerebrovascular conditions, neurosurgical trainees in bypass surgery are declining in numbers. Microsurgical suturing skill can only be achieved through regular practice. Classical microvascular anastomosis training models incorporate suturing of gloves, cadaveric vessels, and synthetic tubes [3]. The trend of microneurosurgery practice in the laboratory has gradually changed to turkey and chicken wing models [4]. Although the use of live animals requires strict institutional protocol regarding animal welfare and ethical issues, live animal models provide the most realistic knowledge of bypass surgery [5].

## Technical report

### Materials and methods

Materials

1. Microscope with between 20 and 40X magnification, 2. Tissue dissecting scissors (Figure A1), 3. Needle applying forceps (Figure A2), 4. Curved smooth tip vessel dilator (Figure A3), 5. Thumb pins, 6. Methylene blue or Pioctanin dye, 7. Contrast micro silicon sheets, 8. A pair of fine pointed micro forceps (Figure A4), 9. 10-0 monofilament non-absorbable round body nylon suture, 10. Bayoneted microscissors for suture cutting (Figure A5).

Methods

The most basic technique involves a microvascular end to end progressed by the end-to-side anastomosis and side-to-side anastomosis in low socioeconomic easily available vascular models like an umbilical cord and chicken wings.

**Figure 1:**
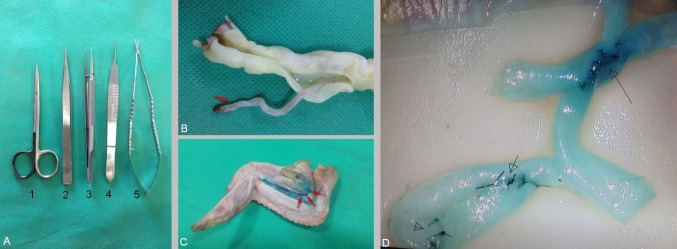
Vascular models A: Basic microinstruments required for practicing anastomosis B: Umbilical artery (red arrow) dissected from the umbilical cord C: Exposure of brachial artery (red arrows) in chicken wing D: Demonstration of an end-to-end anastomosis (open arrowhead), side-to-end (small arrow) and side-to-side anastomosis (long arrow) in brachial artery harvested from the chicken wing under 30X magnification. Interrupted sutures are placed with 10-0 nylon sutures

Vessel isolation: Fresh specimen is ideal for practice. Umbilical artery (single red arrow) is harvested from the umbilical cord by stripping off the epithelium and Wharton’s jelly (Figure 1B). Brachial artery (BA) is the choice of an artery in chicken wings. Artery dissection starts from the skin, where the skin is lifted up and dissected from the ventral side from the shoulder towards the tip of the wing. BA is identified between the biceps brachii and triceps brachii along the shaft of the humerus (Figure 1C three red arrows). Harvested length is around 4 to 5 cm long with approximately 1 mm diameter in size which is identical to the cortical vessel of the human brain.

Vessel preparation: Under the microscope, stump preparation is started by meticulous dissection to harvest the full thickness artery leaving adventitia intact. Connective tissue around the stump is removed. Sufficient exposure is needed for comfort space to repair. Continuous irrigation is necessary to avoid vascular tension, kinking, and twisting. The artery is then divided for practice. Methylene blue or Pioctanin (violet dye) is colored cautiously around the opening with the help of microforceps tip. A piece of contrast silicone sheet is placed beneath the two ends.

Arteriotomy: Transverse slit arteriotomy is either done with 11 number pointed blade or sharp pointed microscissors to create a hole in the recipient's vessel for side-to-end and side-to-side anastomosis practice. Forceps should be atraumatic to prevent crushing of intima and media (Figure 1D).

Suturing method: Preferred sutures are continuous and interrupted sutures. 10-0 or 9-0 monofilament nylon is used. Ideal length of the suture should not exceed 5 cm for better visualization of entire length within the operative field. Interrupted sutures are preferred if the size of the recipient and donor is mismatched and the chance of tearing down the vessel or purse stringing is anticipated. Microscope handling is another essential step. Zoom-in and zoom-out are repeatedly practiced. While taking up bites, microscope should be zoomed in, and after completing tying knots, it should be zoomed out and cut. Needle insertion space from the stump edge should be of the same thickness of the vessel and distance between two sutures should be as twice as the thickness of the vessel. The adventitial layer is lightly grasped. Provide a counter pressure when the needle is passed through the wall. Bites are taken to transverse media and intima. Some surgeons preferred to transverse all three layers. Bites are taken inside-out in recipient artery and outside-in in donor side. Sutures are evenly spaced to allow distribution of tension equally on the anastomosis zone. Threads are pulled gently in one direction to avoid cuts through the arterial wall. Flow patency is adjusted by injecting saline in and out prior to the completion of the anastomosis.

End-to-end anastomosis: Artery is divided at a healthy point. An appropriately sized graft is usually favored. In the case of size disparity, either a recipient or a donor end is beveled to match the size. Beveling technique is helpful to enlarge the diameter of the anastomosis to avoid purse stringing and stenosis. Posterior wall suture is taken as a first bite followed by interrupted sutures. Results are superior when sutures are placed inside out in the direction of blood flow during stitching the two ends together. Edges of the anastomosis should be everted (eversion technique) to match the inner layer of the stump and minimize narrowing of the lumen that is caused by the sutures. Minimum of six and maximum of 10 throws are required as per the diameter of the lumen on one side (Figure 1D, open arrowhead).

End-to-side technique: Heel and toe of the graft are cut in a beveled fashion at 60° angle resembling a fish mouth. The same length is incised down the vessel end from the inferior corner to widen the stump opening. The length of the arteriotomy should be 1.5 times the graft diameter. The graft is then cut to match the length of the arteriotomy. Suturing begins at the heel. Heel and toe are the most critical points to handle. Suturing is started two or three bites from the apex of the heel. Bites are taken outside-in fashion in donor's vessel and inside-out in the recipient. Two to three throws are taken on either side of the apex before the heel of the graft is brought down to the artery. Anastomosis is completed by continuing either running or interrupted sutures down the side around the toe. Suturing is continued to the contralateral side until it meets the starting point (Figure 1D, short arrow). Interrupted sutures are placed in such a way as to feed the laying-off donor vessel wall towards the toe of the anastomosis. Poorly placed sutures may either produce narrowing or bleed through the anastomosis.

## Discussion

Development of microsurgical anastomosis has allowed complex reconstructive surgical procedures. Surgical residents are required to have enhanced technical knowledge by practicing with different kinds of vascular models. Time invested in practicing yields a high level of confidence when one starts operating in theatre. Among other subspecialties, vascular neurosurgery is independent and challenging, where surgeons are required to have enhanced technical knowledge for optimal treatment of the cerebrovascular diseases. Adequate training to young neurosurgeons should be best aligned to augment the empowerment of surgical dexterities and practical understanding that helps to build microsurgical skills. Increasing use of neurosurgical techniques necessitates intensive lab training in microsurgery [6]. The most basic technique involves microvascular end-to-end upgraded by end-to-side anastomosis. Sufficient opportunity in routine surgery may not be available for the beginners. Many training models such as suturing of surgical gloves, silastic tubes, and animal models can be used for learning microvascular technique [4,7]. This kind of practice also helps senior vascular surgeons to maintain and sharpen the skills. In current training courses live rats are mostly used to acquire ultimate competence in microvascular anastomosis as an advance course.

Bimanual dissection is better than a single hand technique while handling micro instruments and they should be handled in such a way that tips should be well visualized. The hand grip is a very important factor for proper instrument handling. It can be classified as power and precision grip [8]. The most commonly used is pen grip with greater stability. Thumb, index and middle fingers are used as a tripod with the ulnar border of hand wrist and the elbow is well supported. Middle finger should rest firmly on the working surface or on a ring finger. Hand flexors and extensors are relaxed avoiding fatigue while the intrinsic muscles perform accurate movements. Physiological tremors usually arise from the mechanical and neuromuscular sources. Tremors are made worse by fatigue, anxiety, excessive intake of alcohol, caffeine, smoking, long hour procedure, and hypoglycemic condition. This can hamper a surgeon’s ability to learn the microsurgical technique. Narrow and deep-seated operative corridor is another factor that produces tremor. A precision hand grip can prevent tremor. The smaller size and light instruments are better for microsurgery. The quiet hand technique is performed by the thumb and I, II, III fingertips. Each muscle contraction adds tiredness and predisposes to tremor [8-10]. Any kind of fixed support can be placed close to the operative site for hand support. Non-dominant hand is able to play a more active role during practice [11].

Although a number of non-suturing techniques have been in clinical and experimental practices, their effectiveness is still lacking [12]. Microvascular anastomosis has been accepted as the most dependable method for revascularization and flows augmentation in neurosurgery. End-to-end and end-to-side techniques are mostly done by vascular neurosurgeons. Both the techniques are performed with simple interrupted sutures. A slight oversized slit can be used to stretch the donor side in end-to-side technique. Due to the presence of inherent elastic recoil, anastomosed area stays patent and may form elliptical shape after assuming blood flow. End-to-side anastomosis is used for bypass procedures for the thrombo-occlusive disease. Two common techniques are used depending on the size of the vessels involved for anastomosis. Open technique is also known as parachute or open “Y” technique. This procedure provides wider ends, thus, increases patency and facilitates anastomosis. The end-to-side procedure is a preferred technique to preserve blood flow distal to the anastomosis, to manage size discrepancy and to avoid vascular spasm [9,12]. Closed technique is easier than open procedure as it is usually performed in larger vessels. Heel and toe are secured with a box stitch. Throws are then continuously given in quadrants bringing down suture from the heel and toe respectively on either side to meet at the midpoint of the arteriotomy. The side-to-side technique can also be indicated either for emergency or elective procedure for cerebral blood flow augmentation or revascularization. Anastomosis is usually chosen for flow replacement after clipping of a complex aneurysm, thus, preventing small vessel occlusion or stenosis. This procedure is basically done during clipping of complex anterior communicating aneurysm or in posterior circulation aneurysm [1]. Side-to-side approximation without creating tension or stenosis at the anastomosis site in side-to-side procedure carries potential technical difficulty.

## Conclusions

Fine microinstruments, hand-eye coordination, visual feedback, and precise hand manipulation are essential aspects for effective anastomosis. It poses various advantages in sharpening the technical skill on anastomosing smaller size of vessel identical to cortical branches of middle cerebral artery. Low-cost laboratory training protocol could be included to boost the empowerment of the surgical dexterities and a basic understanding of microneurosurgery during residency, particularly in source restraint areas.
